# African swine fever virus MGF505-3R facilitates ferroptosis to restrict TBK1-IRF3 pathway

**DOI:** 10.1128/spectrum.03423-24

**Published:** 2025-06-23

**Authors:** Sai Niu, Ying Zhou, Chunyue Fang, Yonggen Yang, Junjie Wang, Shandian Gao, Hanchuan Dai

**Affiliations:** 1 College of Veterinary Medicine, Huazhong Agricultural University 627716, Wuhan, Hubei, China; 2 Lanzhou Veterinary Research Institute, Chinese Academy of Agricultural Sciences, Lanzhou, Gansu, China; Xinxiang Medical University, Xinxiang, China

**Keywords:** African swine fever virus, MGF505-3R, GPX4, ferroptosis, IRF3

## Abstract

African swine fever virus (ASFV) causes hemorrhagic, severe infectious diseases and serious economic losses to the pig industry. ASFV multigene family 505 can antagonize the host’s innate immunity through multiple signaling pathways and is considered an important target for vaccine development. However, the mechanism by which it induces host cell damage remains unclear. In this study, we observed that ASFV infection, similar to RSL3, can induce ferroptosis with the accumulation of reactive oxygen species (ROS) and iron, decrease glutathione peroxidase 4 (GPX4) expression, and restrain the Kelch-like ECH-associated protein 1-nuclear factor E2-related factor (Keap1-Nrf2) pathway. Moreover, the expression of ferroptosis biomarkers (LOX and PTGS2) has been moderately upregulated. Some proteins related to ASFV replication, invasion, and infection were evaluated for evidence of ferroptosis. MGF505-3R interacts with GPX4 to undergo ferroptosis, resulting in ROS accumulation, mitochondrial membrane potential destruction, and NCOA4-mediated ferritinophagy elevation. In addition, MGF505-3R suppressed the Keap1-Nrf2 pathway, while GPX4 activation counteracted its stimulatory effect on TANK-binding kinase 1 (TBK1)-IRF3 phosphorylation. Importantly, the transcription levels of interferon beta (IFN-β), ISG15, and ISG54 were elevated after GPX4 activation, suggesting that ferroptosis resistance could reverse the inhibition of the TBK1-IRF3 pathway and IFN-β levels induced by MGF505-3R. These findings provide new ideas and directions for elucidating the mechanism of ASFV-induced oxidative damage and lay a significant foundation for revealing the pathogenic mechanism of the virus by targeting ferroptosis.

**IMPORTANCE:**

We revealed that ASFV infection and MGF505-3R transfection induced the accumulation of iron and ROS, resulting in NCOA4-mediated ferritinophagy and ferroptosis, as well as restricted GPX4 expression and the Keap1-Nrf2 pathway. GPX4 activation promotes the TBK1-IRF3-IFN-β pathway and exerts antiviral activity. These findings indicate that ASFV facilitates ferroptosis, providing a proof of principle that may be applicable to oxidative damage and lipid peroxidation manipulation-based therapy for ASFV infection. Given the GPX4 downregulation in ASFV infection, GPX4 activation and ferroptosis resistance highlight its potential as a therapeutic target for viral infection.

## INTRODUCTION

African swine fever is a severe and highly contagious hemorrhagic disease caused by African swine fever virus (ASFV), which infects domestic pigs and wild boars. Currently, there are no effective vaccines against ASFV. The ASFV genome contains several distinct multigene families (MGFs): MGF-100, MGF-110, MGF-300, MGF-360, and MGF-505, which have been originally identified as repetitive sequences in terminal genomic regions and named based on the average length of their predicted gene products (1, 2). MGF505 is located in the highly variable left-terminal genomic region, encodes products with structural similarities (3, 4), and is important for ASFV replication in cells (5). MGF505 genes have been reported to evade host innate immunity through JAK-STAT, cGAS-STING, and RIG-I-MAVS (6, 7) and are considered potential targets for the development of ASFV vaccines. Furthermore, MGF505-3R participates in the inhibition of interferon (IFN) production. Deletion of MGF505-3R and other MGF genes results in weakened virulence, reduced virus replication, and attenuation of the virus in pigs (8). However, the mechanism by which MGF505-3R induces immunosuppression in host cells requires further investigation.

Viral infection-mediated immune evasion and innate immunity suppression are mechanistically linked to redox imbalance, which may originate from dysregulated iron metabolism. Iron is an essential trace element for cellular activities. The iron metabolism dysfunction-induced Fenton reaction produces a large number of lipid free radicals that attack the cell membrane lipid bilayer, thus causing cellular oxidative damage and cell death (9, 10). Ferroptosis is iron-dependent regulated cell death caused by massive lipid peroxidation-mediated membrane damage (11). LOX and PTGS2 act as sensitive biomarkers of ferroptotic activity, with their upregulation linked to increased ferroptotic susceptibility (12). Ferroptosis inhibits the import of cysteine, resulting in glutathione (GSH) depletion and consequent inactivation of phospholipid peroxidase glutathione peroxidase 4 (GPX4) (11, 13). Most viral infections that invade host cells replicate and are released during ferroptosis, causing host tissue/organ damage and facilitating viral spread. Studies have shown that hepatitis B virus reduces ferroptosis by reducing intracellular Fe^2+^ levels and activating GPX4 expression through the SRSF2/PCLAF axis (14). Swine influenza virus can trigger a reduction in the Xc^−^/GPX4 axis, resulting in GSH depletion to enhance viral replication (15). Infection with Epstein-Barr virus (EBV) can induce cellular GPX4 to promote drug resistance in nasopharyngeal carcinoma (16). However, studies on ASFV infection in ferroptosis are seldom reported.

Multiple signaling pathways contribute to the regulation of intracellular redox levels. The Kelch-like ECH-associated protein 1-nuclear factor E2-related factor (Keap1-Nrf2) pathway is a prospective therapeutic target (17). Under homeostatic conditions, Nrf2 degradation is promoted when Keap1 interacts with Nrf2. Under conditions of oxidative stress or exposure to reactive oxygen species (ROS), Keap1 is modified by cysteine residues to inhibit degradation mediated by Keap1, which facilitates Nrf2 nuclear translocation to bind antioxidant response elements in the 5′ upstream promoter region (18, 19), ultimately increasing the transcription of Nrf2-dependent genes, thereby elevating the expression of antioxidative enzymes, such as heme oxygenase-1 (HO-1), NADPH quinone oxidation reductase-1, superoxide dismutase (SOD), glutathione S-transferase, and γ-glutamylcysteine ligase (20), and subsequently exerting cytoprotective antioxidative function (21). Many viruses can restrict the Keap1-Nrf2 cascade to exacerbate lipid peroxidation and ferroptosis. Herpes simplex virus type 1 (HSV-1) infection elevated Nrf2 ubiquitination and degradation, which resulted in substantial reduction in the expression of antiferroptotic genes downstream of Nrf2, thus disturbing redox homeostasis and facilitating ferroptosis (22). Influenza A virus (IAV) (H1N1) can induce ferroptosis in human nasal epithelial cells via the Keap1-Nrf2-GCLC signalling pathway (23). Bovine viral diarrhea virus infection inhibits cytoplasmic and mitochondrial GPX4 expression via the Nrf2-GPX4 pathway by degrading ferritin via NCOA4-mediated ferritinophagy to elevate Fe^2+^ accumulation and facilitate ferroptosis (24). These findings shed new light on the physiological effects of ferroptosis on the pathogenesis of viral infections and provide a promising therapeutic strategy for infectious diseases.

In this study, we determined the involvement of ferroptosis and Keap1-Nrf2 signaling in porcine alveolar macrophage (PAM) cells infected with ASFV, and the role of MGF505-3R in ferroptosis, Keap1-Nrf2 signaling, and interferon beta (IFN-β) production was subsequently investigated. Our data revealed that ASFV infection induced ferroptosis with iron accumulation in PAM cells and that Keap1-Nrf2 signaling was restricted. MGF505-3R suppresses GPX4 expression and blocks Keap1-Nrf2 signaling, thereby restraining IFN-β expression. Notably, GPX4 activation effectively reverses these processes. These findings not only advance our understanding of ASFV-induced oxidative damage mechanisms but also provide crucial insights into viral pathogenesis.

## MATERIALS AND METHODS

### Cells and virus

PK-15, HEK-293T, and PAM cells were maintained in Minimum Essential Medium, Dulbecco’s Modified Eagle Medium, and Roswell Park Memorial Institute 1640 medium, respectively (Gibco, USA). All media were supplemented with 10% fetal bovine serum (Gibco) and 1% penicillin-streptomycin solution (Gibco), and the cells were cultured at 37°C with 5% CO_2_. PK-15 and HEK-293T cells were stored in our laboratory, and PAM cells were isolated according to the World Organization for Animal Health’s porcine primary cell isolation method. Cells were seeded in 6- or 12-well plates (Corning, USA) at 1 × 10^5^ or 1 × 10^4^ cells/well and then transfected using Hieff Trans Liposomal Transfection Reagent (Yeasen, China), according to the manufacturer’s instructions, with the plasmids at a confluence of 70%–80%. ASFV SY18 strain (GenBank accession number MH766894) was propagated in PAM cells as previously described (25). Viral titers were determined by calculating the log_10_ 50% tissue culture infectious dose per milliliter of PAM cells. PAM cells were washed with phosphate buﬀer solution (PBS) and exposed to ASFV at a multiplicity of infection of 1.0. The cells were collected at the indicated time points after infection. All experiments with the ASFV virus were performed in a biosafety level 3 laboratory.

### Plasmid construction

The plasmids used in this study were as follows: the MGF505-3R gene of the ASFV SY18 strain was cloned into the *pCAGGS-Flag* or *pcDNA3.1-EGFP* vector using homologous recombination with *EcoR I*, *Hind III*, *Nhe I*, and *Xho I* (Thermo Scientific, USA). Plasmids encoding the MGF505-1R, MGF505-7R, A151R, A179L, A224L, S273R, F334L, F778R, D1133L, and P1192R gene fragments of the ASFV SY18 strain virus were constructed using homologous recombination amplification. Primers (synthesized by Sangon Biotech, China) are listed in Table 1. The plasmids were transfected into HEK293T cells using Lipo3000 (Beyotime, China), and subsequent experiments were performed 24 h post-transfection.

**TABLE 1:** Primers for gene homologous recombination amplification

Primer name	Sequence (5′−3′)
MGF505-1R-EGFP-F	CGTTTAAACTTAAGCTTATGTTCTCTCTCCAGAACTTATGTCG
MGF505-1R-EGFP-R	GTCGACTGCAGAATTCAGGCATGTATTGTTTAGAATAAATGG
MGF505-3R-EGFP-F	CGTTTAAACTTAAGCTTATGTCCTCTTCTCTTCAGGAACTTTG
MGF505-3R-EGFP-R	TACCGTCGACTGCAGAATTCGCTTTCCCAGGTTTCGAAGTATT
MGF505-3R-FLAG-F	GCTAGCATGTCCTCTTCTCTTCAGGAAC
MGF505-3R-FLAG-R	AATTCCGCTTTCCCAGGTTTCGAAGTAT
MGF505-7R-EGFP-F	GAGACCCAAGCTGGCTAGCATGTTCTCCCTTCAGGACCTCTG
MGF505-7R-EGFP-R	CGTCGACTGCAGAATTCATACATGGCATACTCCAAAGCATAG
A151R- EGFP-F	AGACCCAAGCTGGCTAGCATGGCGTTGTTACACAAAGAAAAG
A151R- EGFP-R	GTACCGTCGACTGCAGAATTCTTGGAATATATTGGGCGACAT
A179L- EGFP-F	TTTAAACTTAAGCTTTATCAAATTGCAGTTTCTTAATAACTGT
A179L- EGFP-R	GTACCGTCGACTGCAGAATTCATGGAGGGAGAAGAGTTAATA
A224L- EGFP-F	GACCCAAGCTGGCTAGCAAGTTTGACACTAAAATTCAGTGTT
A224L- EGFP-R	GTACCGTCGACTGCAGAATTCATGTTTCCTAAAATAAATAC
S273R- EGFP-F	AACTTAAGCTTATGTCTATATTAGAAAAAATTACGTCAAGTCC
S273R- EGFP-R	GTACCGTCGACTGCAGAATTCTGCGATGCGAAACAGATGG
F334L- EGFP-F	TTTAAACTTAAGCTTTTAAAAATCATCGTCGTTTAAAAAGAG
F334L- EGFP-R	GTACCGTCGACTGCAGAATTCATGTTAATATTTATTTCAAATA
F778R- EGFP-F	GACCCAAGCTGGCTAGCATGGAGACGTTTTTTATTGAGACGT
F778R- EGFP-R	GTACCGTCGACTGCAGAATTCCAGAAGACACGCCTCGCA
D1133L- EGFP-F	GGAGACCCAAGCTGGCTAGCAGATGTGTGCACAAGTGACGG
D1133L- EGFP-R	GTACCGTCGACTGCAGAATTCATGGCGTATCCCGAATTGG
P1192R- EGFP-F	TAGCGTTTAAACTTAAGCTTATGGAAGCGTTTGAAATCAGCG
P1192R- EGFP-R	TCCGAGCTCGGTACCAAGCTTATGAAATTTCCACTGAGTATTT

### RNA extraction and quantitative PCR

Total RNA was extracted from cells with TRIzol reagent (Invitrogen, Carlsbad, CA, USA), and 1 µg of RNA was reverse transcribed to cDNA using Hifair II 1st Strand cDNA Synthesis SuperMix for quantitative PCR (qPCR) (gDNA digester plus) (Yeasen). To determine the effects of MGF505-3R on the expression of IFN-β, ISG15, and ISG54, PK-15 cells were transfected with 2.5 µg of either the pCAGGS empty vector or a plasmid encoding the MGF505-3R. After 12 h, the cells were treated with poly(deoxyadenylic acid:deoxythymidylic acid) [poly(dA:dT)] for 12 h. qPCR was performed using qPCR Supermix (Yeasen) on a Bio-Rad CFX96 real-time system. The abundance of individual mRNA transcripts was measured in triplicate and normalized to glyceraldehyde-3-phosphate dehydrogenase (GAPDH) mRNA using the 2^−ΔΔCt^ method. All samples were analyzed in triplicate. Primers (synthesized by Sangon Biotech) are listed in Table 2.

**TABLE 2:** The primer sequence of qPCR

Gene	Sequence (5′−3′)	GenBank ID
IFN-β	F: TGCAACCACCACAATTCCAGAAGG R: TGACGGTTTCATTCCAGCCAGTG	NM_002176.4
ISG15	F: CCCTTGAGGGACTGCATGAT R: GACCCTTGTCGTTCCTCACC	NM_001128469.3
ISG54	F: GCACAGCAATCATGAGTGAGAC R: GCTTGCCGTAAGCATTCCAG	NM_001315658.1
GAPDH	F: ACCCAGAAGACTGTGGATGG R: ACGCCTGCTTCACCACCTTC	NM_001256799.3
B646L	F: AGTTATGGGAAACCCGACCC R: CCCTGAATCGGAGCATCCT	MW736605.1
EP402R	F: atgaagaagaacaatgtcagca R: actgataacgactgtaaggct	OQ164539.1
GPX4	F: TGTGTGAATGGGGACGATGC R: CTTCACCACACAGCCGTTCT	NM_214407.1
Keap1	F: TACAACCCCAGTGATGGCAC R: GACCCCAACCCCAATTCGAT	NM_001114671.1
Nrf2	F: CTACGGGATTGGGGTTTGGG R: CCCCGTGACTAGGCACATTT	XM_013984303.2
HO-1	F: TACCGCTCCCGAATGAACAC R: GTCACGGGAGTGGAGTCTTG	NM_001004027.1

### Molecular docking analysis of MGF505-3R and GPX4

To investigate the interaction between A151R and GPX4, the HDOCK online program (http://hdock.phys.hust.edu.cn/) was used to dock the structures of MGF505-3R (PDB ID: seven cma) and GPX4 (PDB ID: 2obi). The program employed a fast Fourier transform algorithm to search for potential binding modes and evaluate the sampled binding modes using a knowledge-based scoring function. The system then returned the top 10 docking results for visualization and the top 100 docking results for download. The docking combination with the most negative docking score was selected, and the interactions were analyzed using PyMOL for visualization.

### Coimmunoprecipitation and Western blotting

Cells were lysed in radioimmunoprecipitation assay (RIPA) buffer (Biosharp, China) containing a protease inhibitor cocktail (Servicebio, China) for 30 min on ice. Protein A + G agarose beads (50 µL) (Yeasen) were incubated with the indicated antibodies at 4°C overnight. The cell lysate was added and incubated for another 6 h at 4°C. The agarose beads were subsequently washed three times with RIPA buffer containing a protease inhibitor cocktail. Protein concentrations were measured using a bicinchoninic acid protein assay kit (Aidlab, China). Proteins were separated by 12% polyacrylamide gel electrophoresis (PAGE) containing 0.1% SDS and transferred to polyvinylidene fluoride membranes. The membranes were incubated for 2 h at room temperature in blocking buffer and probed with antibodies at 4°C overnight. After washing three times with Tris-buffered saline and Tween 20, the membranes were incubated with horseradish peroxidase (HRP) at room temperature for 2 h. GAPDH was used as an internal control. The reacted proteins were visualized using an electrochemiluminescence system (Tanon, China). Antibodies used in this study were as follows: anti-Flag tag antibody was purchased from Proteintech, China. Anti-IRF3 and TANK-binding kinase 1 (TBK1) polyclonal antibodies were purchased from CST, USA. Anti-TfRC, NCOA4, GPX4, FTH, Nrf2, and Keap1 polyclonal antibodies were purchased from Abclonal, China. Phospho-TBK1 polyclonal antibody was purchased from Affair Biotech, USA. Phospho-IRF3 polyclonal antibody was purchased from Yubo Biotechnology, China. GAPDH antibody and antimouse and antirabbit IgG HRP secondary antibodies were purchased from Servicebio. Antimouse and antirabbit IgG 488 green fluorescence and 594 red fluorescence secondary antibodies were purchased from Proteintech.

### Immunofluorescence assay

PK-15 cells were cultured in 6- or 12-well plates and transfected with either 3R-Flag or empty vector. Twenty-four hours later, the cells were transfected with or without poly(dA:dT). After 12 h, cells were harvested and fixed with 4% paraformaldehyde at 37°C for 20 min. Next, the cells were incubated with PBS containing 1% bovine serum albumin and 1% Triton X-100 at 37°C for 2 h and followed by incubation with primary antibodies overnight at 4°C. After three 30 min washes, the cells were further incubated with Alexa Fluor 488-conjugated Affinipure goat antirabbit IgG (H + L) or Alexa Fluor 594-conjugated Affinipure goat antimouse IgG (H + L) for 1 h at room temperature. Then, the cells were examined using a confocal microscope (model LSM880; Zeiss, Germany).

### Liquid chromatography-tandem mass spectrometry analysis

A coimmunoprecipitation assay was performed, and the precipitated proteins were subjected to SDS-PAGE. The gels were silver stained and sent to Novogene (Beijing, China) for liquid chromatography–tandem mass spectrometry (LC-MS/MS) analysis to identify the potential interaction protein between MGF505-3R and the host protein. The protein samples were digested into peptides, which were then injected into a C18 nanotrap column (26). An analytical column was used with the column oven temperature set to 55°C. Peptide separation was followed by analysis using an Orbitrap Exploris 480 mass spectrometer equipped with FAIMS (Thermo Fisher), with a Nanospray Flex electrospray ionization source operating at 2.1 kV spray voltage and 320°C ion transport capillary temperature. The scan-round time in tandem mass spectrometry was set to 1 s, and the precursors in the full scan were selected from high to low abundance and fragmented by higher-energy collisional dissociation; the automatic gain control target value was 7.5 × 10^4^; the maximum ion injection time was 22 ms; the normalized collision energy was set to 30%, the intensity threshold was 5.0 × 10^3^, and the dynamic exclusion parameter was 40 s.

### The functional analysis of protein

Gene Ontology (GO, https://www.geneontology.org/) was used to analyze the cellular components, molecular functions, and biological processes of the peptides obtained by mass spectrometry. InterPro (IPR, https://www.ebi.ac.uk/interpro/) functional analyses were conducted using the interscan program against a non-redundant protein database and domain annotation of functionally unknown proteins using pattern structures or features. The Clusters of Orthologous Groups (https://www.ncbi.nlm.nih.gov/research/cog-project/) and Kyoto Encyclopedia of Genes and Genomes (KEGG, https://www.genome.jp/kegg/) databases were used to analyze the protein families and pathways.

### ROS and mitochondrial membrane potential measurement

The 2′,7′-dichlorodihydrofluorescein diacetate (DCFH-DA) fluorescent probe (Dojindo Laboratories, Kumamoto, Japan) was used to detect intracellular ROS levels. PK-15 cells (2 × 10^4^ cells/well in a 12-well plate) were transfected for 24 h. Then, the cells were rinsed three times with PBS before the DCFH-DA fluorescence probe was loaded for 30 min at 37°C. Mitochondrial membrane potential (MMP) was assessed using JC-1 fluorescent dye (Dojindo Laboratories) following the manufacturer’s protocol. After nuclear staining with 4′,6-diamidino-2-phenylindole for 5 min, the cells were washed three times with PBS and imaged with a fluorescence microscope (Olympus IX73, Japan).

### Intracellular GSH and malondialdehyde measurement

The intracellular GSH levels were measured using a GSH assay kit (Beyotime). Cells were treated and cultured in six-well plates, and the samples were subjected to two rapid freeze-thaw cycles using liquid nitrogen and a 37°C water bath. The supernatants were collected by centrifugation and used for GSH measurement. The absorbance at 405 nm was measured using a microplate reader to calculate GSH concentration according to the manufacturer’s instructions. Lipid peroxidation levels were measured using a colorimetric reaction based on the reaction between malondialdehyde (MDA) and thiobarbituric acid, which produces a red product. The absorbance was measured at 450 nm using a microplate reader according to the manufacturer’s instructions (Beyotime).

### Intracellular iron measurement

Intracellular iron concentration was measured using a Total Iron Content Colorimetric Assay Kit (Dojindo Laboratories). Cell samples were processed after differential treatment according to the manufacturer’s instructions, and the iron concentration was calculated by measuring the absorbance in nanometer using a microplate reader. The ferrous iron levels of the unstable intracellular iron pool were determined using a FerroOrange probe (Dojindo Laboratories) according to the manufacturer’s protocol. Images were obtained using a confocal microscope (Olympus IX73).

### Statistical analysis

Data are expressed as mean ± SEM of at least three independent experiments for each experimental group. The significance of the differences between the two groups was assessed using Student’s *t*-test with GraphPad Prism version 8.0. A *P* value of <0.05 was marked as *; a *P* value of <0.01, which means extremely significant difference, was marked as **; a *P* value of <0.001, which means extremely significant difference, was marked as ***; and ns means non-significance.

## RESULTS

### ASFV infection elevates the intracellular iron levels and restricts Keap1-Nrf2 signaling

**Fig 1:**
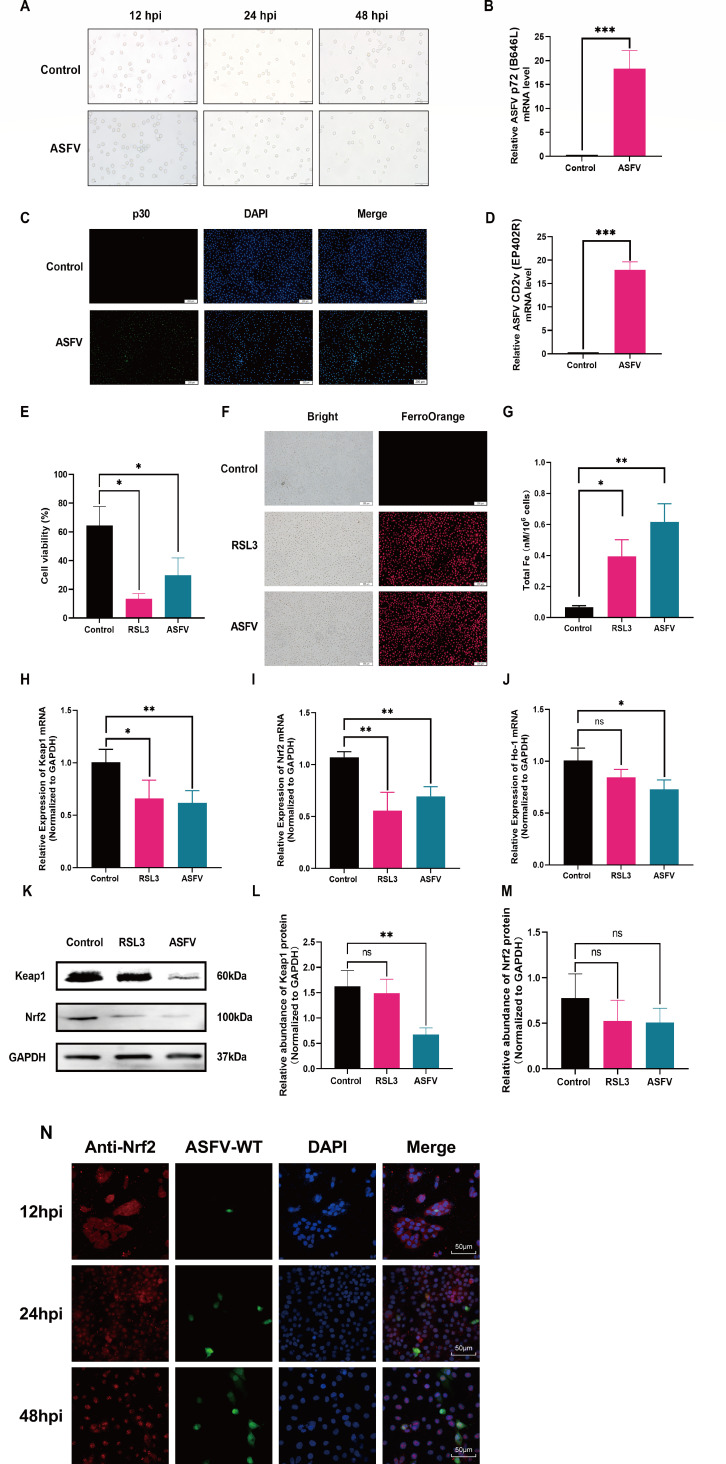
ASFV infection elevates the intracellular iron levels and restricts Keap1-Nrf2 signaling in the PAM cells. PAM cells were treated with ASFV and RSL3. The cells and RNA were harvested and subjected to microscopy, qPCR, or immunofluorescence. (**A**) The observations of the PAM cells under microscope after ASFV infection (multiplicity of infection of 1.0) at 12, 24, and 48 h. The phenomenon of cell death and shedding was obviously observed after treatment for 24 h. The subsequent assays were carried out at 24 h after ASFV treatment. (**B**) p72 mRNA expression. (**C**) p30 protein expression. (**D**) CD2v mRNA expression. (**E**) Cell activity. (**F and G**) Intracellular iron levels. (**H**) Keap1 mRNA expression. (**I**) Nrf2 mRNA expression. (**J**) HO-1 mRNA expression. (**K and L**) Keap1 expression. (**K and M**) Nrf2 expression. (**N**) The dynamic changes of Nrf2 localization. ASFV-WT indirect immunofluorescence of p30 protein. *P < 0.05, **P < 0.01, ***P < 0.001. ns, not significant.

Viral invasion usually induces cellular damage and compromises the antioxidative capacity of the host. To explore whether ASFV infection caused oxidative cell damage, PAM cells were infected with ASFV for 12, 24, and 48 h. The results indicated that ASFV infection caused cell shrinkage, impaired cell growth, and induced significant cell death/detachment after treatment for 24 h (Fig. 1A). The expression of ASFV marker genes or protein, such as p72 (B646L), p30, and CD2v (EP402R), was significantly increased by qPCR and fluorescence microscopy (Fig. 1B through D).

Notably, ASFV infection resisted cell activity, which was similar to that of RSL3 (Fig. 1E). RSL3, a known ferroptosis inducer, directly suppresses GPX4 to facilitate ferroptosis (27, 28). Subsequent analysis of intracellular iron levels using FerroOrange (a fluorescent iron-sensing probe that selectively binds Fe²^+^) demonstrated elevated intracellular Fe²^+^ levels following ASFV infection, consistent with RSL3 treatment (Fig. 1F and G). Furthermore, ASFV infection downregulated the mRNA expression of both Keap1 (Fig. 1H) and Nrf2 (Fig. 1I), and the levels of HO-1 were suppressed (Fig. 1J).

To investigate the dynamic changes of Nrf2 during ASFV infection, PAM cells were infected with ASFV and analyzed at 12, 24, and 48 h post-infection (hpi) using immunofluorescence microscopy. At 12 hpi, during the early infection phase, Nrf2 exhibited a diffuse cytoplasmic localization pattern, primarily concentrated in the perinuclear region. By 24 hpi, Nrf2 was characterized by a shift from a diffuse cytoplasmic distribution to the formation of distinct cytoplasmic aggregates, suggesting initiation of nuclear translocation signaling. At 48 hpi, Nrf2 exhibited complete nuclear translocation with no detectable signal remaining in the cytoplasm. The dynamic changes in Nrf2 correlated temporally with viral replication throughout the infection time course (Fig. 1N). These findings demonstrate that ASFV infection led to Fe^2+^ accumulation and restrained the Keap1-Nrf2 pathway, which subsequently facilitated cell oxidative damage.

### ASFV infection induces ferroptosis in host cells

**Fig 2:**
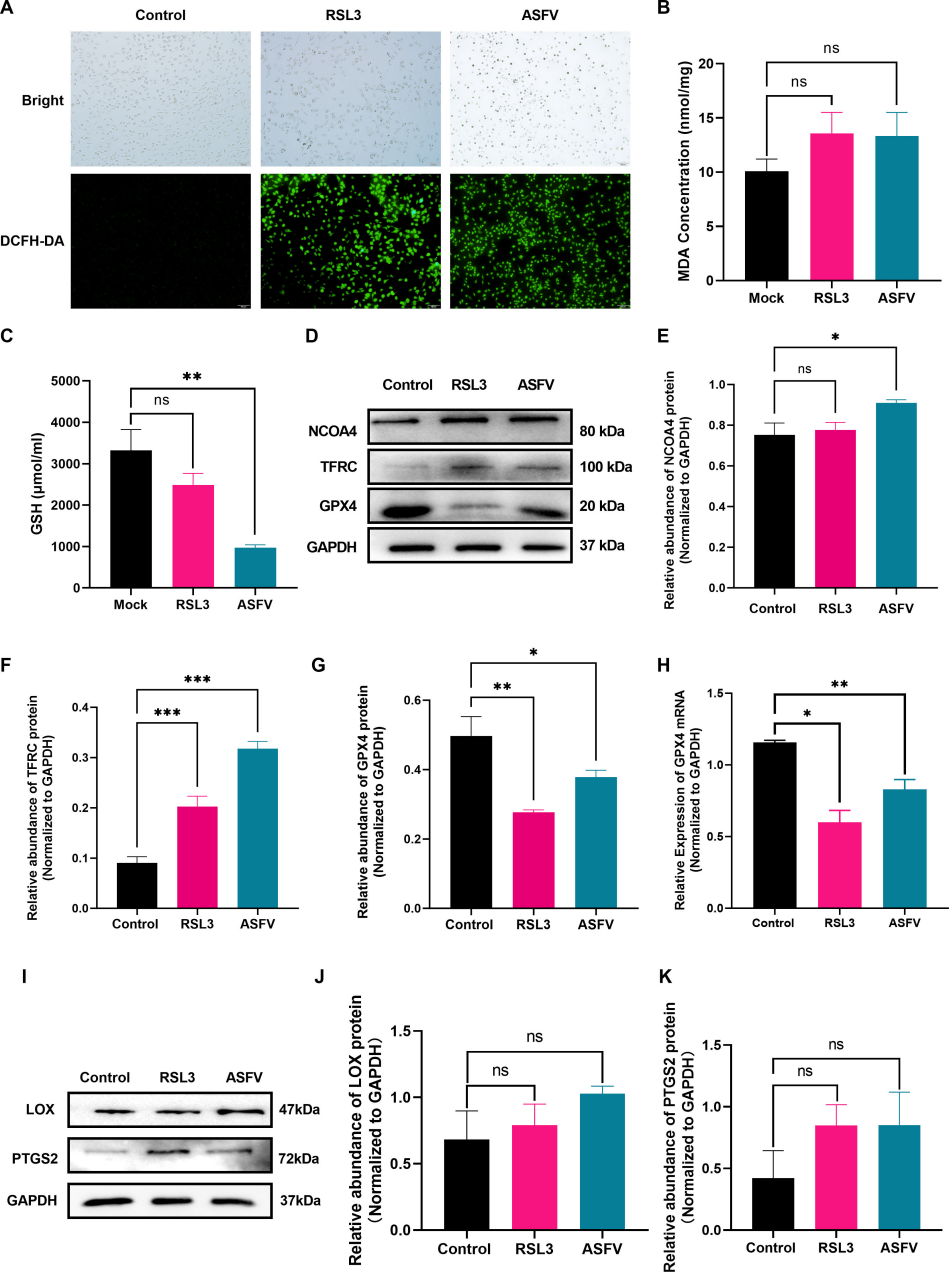
ASFV infection induces ferroptosis in host cells. The PAM cells were treated with ASFV and RSL3 for 24 h. The cells, protein, and RNA were harvested. The samples were subjected to ROS level assay, Western blot, and qPCR. (**A**) ROS accumulation. (**B**) MDA concentration. (**C**) GSH levels. (**D and E**) NCOA4 protein expression. (**D and F**) TFRC protein expression. (**D and G**) GPX4 protein expression. (**H**) GPX4 mRNA expression. (**I and J**) LOX protein expression. (**I and K**) PTGS2 protein expression. *P < 0.05, **P < 0.01, ***P < 0.001. ns, not significant.

Fe^2+^ accumulation contributes to oxidative damage and induces ferroptosis. However, ASFV-induced ferroptosis has rarely been reported. We evaluated evidence of ferroptosis in ASFV-infected PAM cells using RSL3 as a positive control. The results indicated that RSL3 and ASFV stimulation significantly elevated ROS accumulation (Fig. 2A), increased MDA levels (Fig. 2B), and decreased GSH content (Fig. 2C), which demonstrated that ASFV infection led to cellular oxidative damage comparable to RSL3 treatment. Therefore, ferroptosis-related proteins have been investigated. NCOA4 facilitates ferroptosis by participating in ferritin turnover through ferritinopathy. Western blotting demonstrated that the levels of NCOA4 (Fig. 2D and E) and TFRC (Fig. 2D and F) were upregulated after 24 h infection compared to controls, indicating that ASFV infection facilitated NCOA4-mediated ferritinophagy. Notably, ASFV infection significantly reduced GPX4 protein levels (Fig. 2D and G) at 24 hpi, mirroring RSL3 effects and confirming ferroptotic characteristics. Consistent with this, GPX4 mRNA expression was also suppressed (Fig. 2H).

Additionally, ASFV infection moderately upregulates the expression of ferroptosis biomarkers, LOX, and PTGS2 (Fig. 2I). These results demonstrate that ASFV promotes NCOA4-mediated ferritinopathy, potentially increasing cellular susceptibility to ferroptosis.

### Proteins interacting with MGF505-3R are involved in the regulation of GPX4

**Fig 3:**
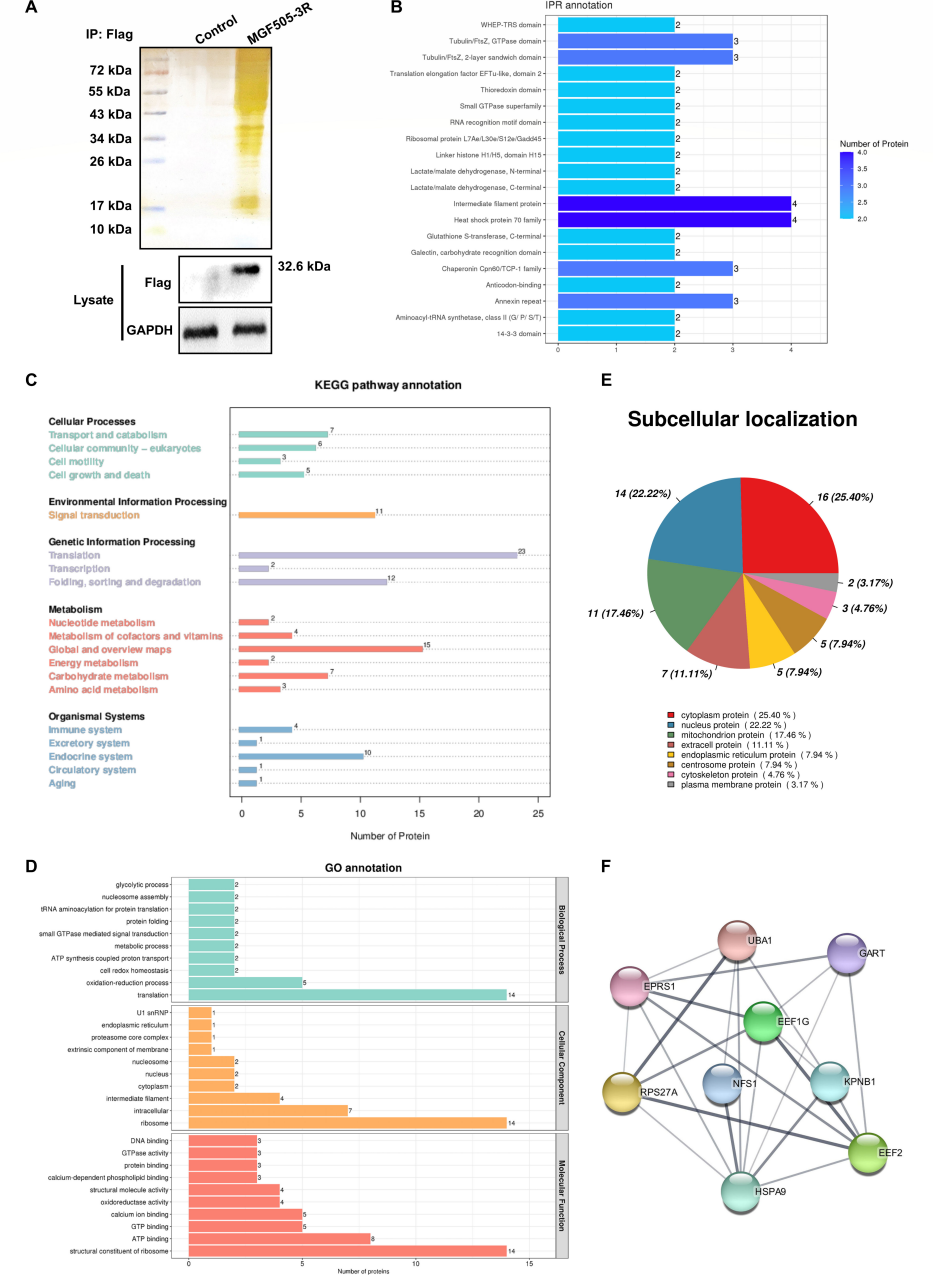
Identification of the proteins interacting with MGF505-3R in PK-15 cells. (**A**) Western blotting and silver staining were used to detect the expression of exogenous MGF505-3R and the enrichment of MGF505-3R interacting proteins in PK-15 cells. Empty Flag vector or Flag-MGF505-3R was transfected into PK-15 cells. Pull down was used with anti-Flag monoclonal antibody 24 h after transfection. MGF505-3R interacting host proteins were eluted from protein A + G agarose gels and analyzed on SDS-PAGE followed by silver staining. GAPDH was used as an internal control. (**B**) Gene ontology (GO) analysis. (**C**) Subcellular localization analysis of interacting proteins. (**D**) InterPro (IPR) database analysis. (**E**) Kyoto Encyclopedia of Genes and Genomes (KEGG) pathway database analysis. (**F**) Drawn MGF505-3R interacting protein grid with Cytoscape and STRING software.

To investigate the exact molecular mechanism of ASFV-induced ferroptosis, we initially screened partial viral proteins for their roles in ASFV-induced ferroptosis. The plasmids of some viral genes (*A151R*, *A179L*, *A224L*, *S273R*, *F334L*, *F778R*, *D1133L*, *P1192R*, *MGF505-1R*, *MGF505-3R*, and *MGF505-7R*) related to virus replication and immune escape were transfected in PK15 cells. All proteins were successfully expressed in the cells. Notably, MGF505-3R significantly suppressed GPX4 expression and elevated TFRC expression (Fig. S1A and B), indicating that MGF505-3R might facilitate ferroptosis and induce the accumulation of ferroportin. Subsequently, proteins interacting with MGF505-3R were identified by coimmunoprecipitation and LC-MS/MS analysis (Fig. 3A). Mass spectrometry results revealed 86 effective peptide segments. GO database analysis revealed that most of them were related to protein translation, ribosome structure, and biological processes (Fig. 3B). Analysis of IPR and KEGG signaling pathway database information showed that MGF505-3R participates in the redox process in host cells. Many proteins, such as redox-related proteins, immune proteins, and catabolic proteins, were precipitated (Fig. 3C through E). The interacting proteins are mainly located in the cytoplasm (Fig. 3E), and we hypothesize these proteins play a role in the stage of cell infection after the virus invades. According to the results of the KEGG database analysis and grid diagram of the proteins interacting with MGF505-3R, many proteins were involved in GSH metabolism (Fig. 3F). For example, GARS is a tRNA ligase composed of glycine; EPRS is a tRNA ligase composed of glutamic acid; and NFS1 is a cysteine desulfurase in the mitochondria. These proteins play biochemical roles in GSH synthesis, and GSH is required for the synthesis of GPX4. Thus, we concluded that MGF505-3R participates in the regulation of GPX4 expression and subsequently induces ferroptosis.

### MGF505-3R interacts with GPX4 to facilitate ferroptosis

**Fig 4:**
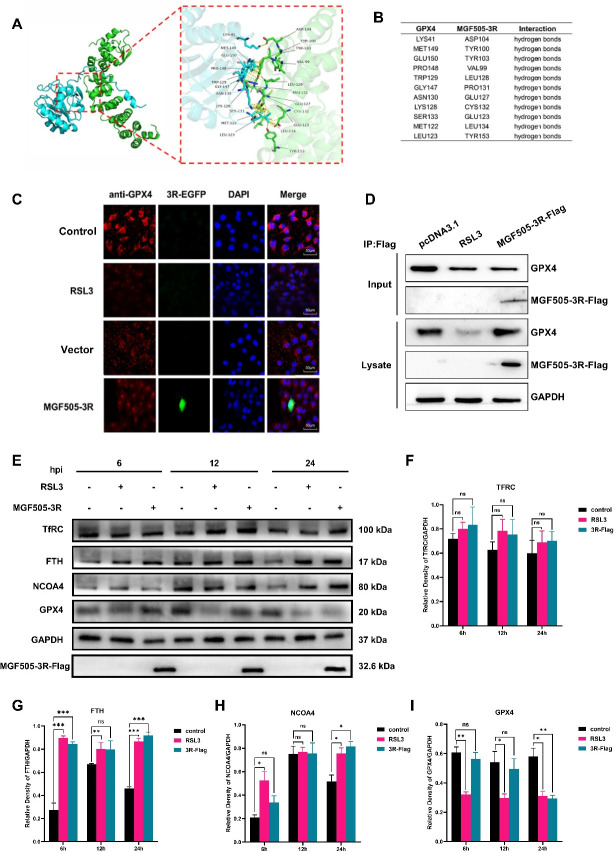
MGF505-3R interacts with endogenous GPX4 to facilitate ferroptosis in the PK-15 cells. Molecular docking was used to analyze the interactions of MGF505-3R and GPX4. The cells were transfected with pcDNA3.1-MGF505-3R-Flag plasmid or treated with RSL3. The cells and proteins were collected. Immunoprecipitation and Western blot were carried out with the indicated antibodies. (**A**) The interaction structure between GPX4 and MGF505-3R was predicted by AlphaFold3 and visualized by Pymol. (**B**) Hydrogen bonding sites were predicted based on the structure by Pymol. (**C**) The colocalization of MGF505-3R and endogenous GPX4. (**D**) The interaction of MGF505-3R and GPX4. (**E and F**) TFRC protein expression. (**E and G**) FTH protein expression. (**E and H**) NCOA4 protein expression. (**E and I**) GPX4 protein expression. *P < 0.05, **P < 0.01,***P < 0.001. ns, not significant.

To investigate whether there was an interaction between MGF505-3R and GPX4, a molecular docking assay was initially conducted. Molecular docking results indicated that MGF505-3R interacted with GPX4 proteins at the atomic level through non-covalent interactions (Fig. 4A), such as hydrophobic interactions (Fig. 4B). To further confirm the interaction between MGF505-3R and GPX4, pCAGGS-MGF505-3R-Flag and pcDNA3.1-MGF505-3R-EGFP were transfected into PK-15 cells and subjected to coimmunoprecipitation and colocalization assays. Laser confocal microscopy revealed that MGF505-3R colocalized with intracellular GPX4 around the nucleus. However, after RSL3 treatment, the fluorescence intensity of GPX4 was significantly reduced (Fig. 4C). Furthermore, the interaction between MGF505-3R and endogenous GPX4 in cells was confirmed by coimmunoprecipitation with anti-Flag and RSL3 (Fig. 4D). These results indicated that MGF505-3R interacted with GPX4. Moreover, it was found that MGF505-3R, similar to RSL3, exerted an inhibitory effect on the expression of the host GPX4 protein. Next, we explored the expression of ferroptosis-related proteins. Further experiments showed that MGF505-3R transfection had no obvious effect on TFRC expression (Fig. 4E and F). However, the levels of FTH (Fig. 4E and G) and NCOA4 (Fig. 4E and H) were elevated after 6, 12, and 24 h, indicating that NCOA4-mediated ferritinophagy was increased. Importantly, consistent with the results of RSL3 treatment, the expression of GPX4 decreased following MGF505-3R transfection. The most significant difference in expression levels was observed 24 h post-transfection (Fig. 4E and H). Taken together, these findings demonstrated that MGF505-3R interacts with GPX4 to facilitate ferroptosis.

### MGF505-3R impairs mitochondrial function and restricts the Keap1-Nrf2 signaling pathway to facilitate oxidative damage

**Fig 5:**
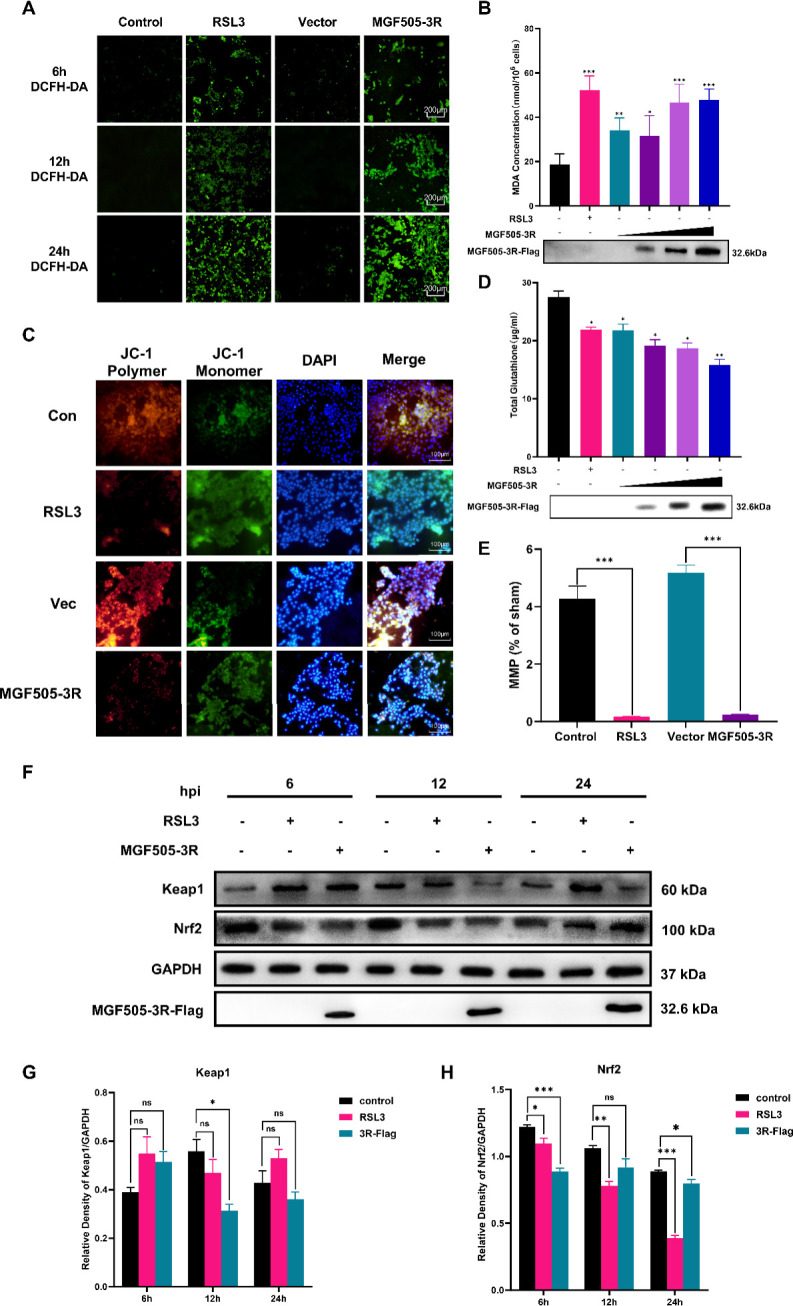
MGF505-3R impairs mitochondrial function and restricts the Keap1-Nrf2 signaling pathway to facilitate oxidative damage. PK-15 cells were transfected with pcDNA3.1-MGF505-3R-Flag plasmid and treated as indicated. Cells and proteins were extracted for analyzing the oxidative damage biomarker and kKeap1-Nrf2 pathway. (**A**) ROS detection by using fluorescent dye DCFH-DA. (**B**) MDA levels. (**C**) GSH levels. (**D and E**) MMP detection by using JC-1 staining. (**F and G**) Keap1 expression. (**F and H**) Nrf2 expression. *P < 0.05, **P < 0.01, ***P < 0.001. ns, not significant.

Viral infection usually results in mitochondrial dysfunction, induced ROS accumulation, and suppressed antioxidative capacity, which might be beneficial for viral replication, proliferation, and immune escape. In this study, ROS accumulation, MMP, MDA, GSH, as well as the Keap1-Nrf2 pathway have been investigated following MGF505-3R transfection. The results of DCFH-DA staining and immunofluorescence microscopy indicated that, similar to RSL3, ROS production increased in a time-dependent manner, and the fluorescence intensity was strongest after transfection for 24 h (Fig. 5A). MGF505-3R transfection increased MDA content (Fig. 5B) and decreased GSH levels (Fig. 5C) in a dose-dependent manner. Furthermore, MGF505-3R administration dramatically decreased MMP levels, which was similar to the results of RSL3 treatment for 24 h (Fig. 5D and E). In addition, the Keap1-Nrf2 pathway was restrained following MGF505-3R treatment. Compared to the control group, Keap1 expression was inhibited at 12 h (Fig. 5E and F). Consistent with the RSL3 treatment, after 6, 12, and 24 h, the expression of Nrf2 protein was decreased (Fig. 5E and G), which might indicate that the binding of Keap1 and Nrf2 was blocked by MGF505-3R, thereby resulting in Keap1-Nrf2 signaling inhibition. These data demonstrate that MGF505-3R promotes ROS accumulation, impairs mitochondrial membrane potential, and restricts the Keap1-Nrf2 pathway, consequently facilitating oxidative damage.

### MGF505-3R blocks the TBK1-IRF3 phosphorylation induced by GPX4 activation to resist IFN-β expression

**Fig 6:**
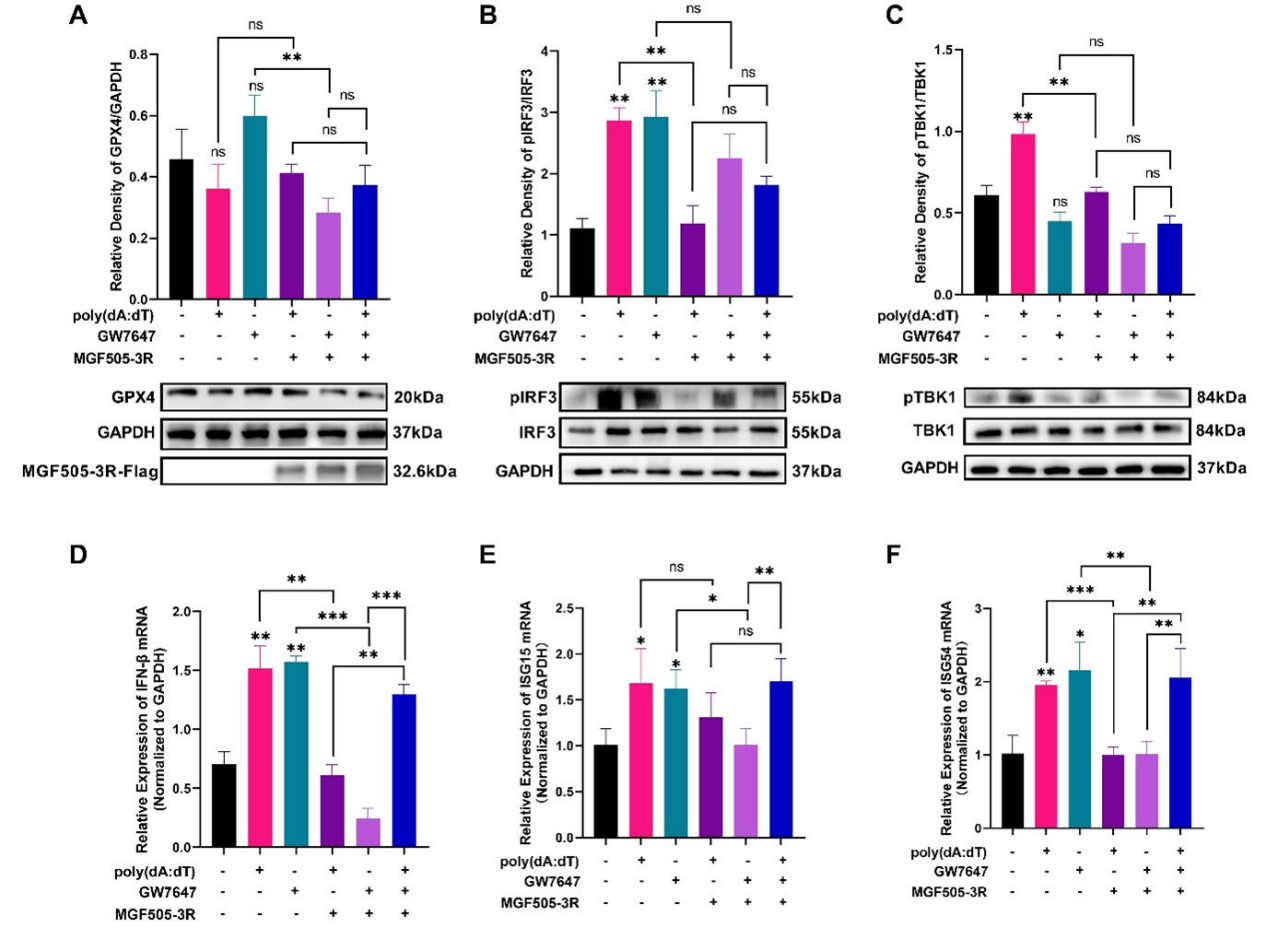
MGF505-3R blocks the TBK1-IRF3 phosphorylation induced by GPX4 activation to resist IFN-β expression. PK-15 cells were transfected with pcDNA3.1-MGF505-3R-Flag plasmid and treated with GW7647 or poly(dA:dT). Protein and RNA were extracted for analyzing the TBK1-IRF3 pathway and IFN-β mRNA expression. (**A**) GPX4 expression. (**B**) pIRF3 and IRF3 expression. (**C**) pTBK1 and TBK1 expression. (**D**) IFN-β mRNA expression. (**E**) ISG15 mRNA expression. (**F**) ISG54 mRNA expression. *P < 0.05, **P < 0.01, ***P < 0.001. ns, not significant.

Viral infections initiate host immune responses through pattern recognition receptors, thereby producing type I IFNs and pro-inflammatory cytokines (29). The TBK1-IRF3 pathway plays a crucial role in IFNs production (30). To investigate how MGF505-3R-mediated ferroptosis affects inflammatory cytokine production and host innate immunity against ASFV, cells were treated with a GPX4 agonist GW7647 (a ferroptosis inhibitor) and poly(dA:dT). GW7647 activates PPARα to upregulate GPX4 expression (31), while poly(dA:dT) induces IFN-β production. GW7647 administration increased GPX4 expression (Fig. 6A) and promoted IRF3 phosphorylation (Fig. 6B) but showed no significant effect on basal IRF3 phosphorylation (Fig. 6C). Poly(dA:dT) treatment significantly increased IRF3 and TBK1 phosphorylation (Fig. 6C). However, MGF505-3R transfection restricted the phosphorylation of IRF3 (Fig. 6B) and TBK1 (Fig. 6C) that was induced by GW7647 and poly(dA:dT). These results show that GPX4 activation and poly(dA:dT) facilitated the IRF3 pathway, which was blocked after MGF505-3R transfection. Subsequently, the transcription of IFN-β and ISGs was explored. The results demonstrated that both GPX4 activation and poly(dA:dT) treatment increased IFN-β (Fig. 6D), ISG15 (Fig. 6E), and ISG54 (Fig. 6F) mRNA levels, which were restricted following MGF505-3R transfection. Notably, these inhibitory effects could be reversed after GW7647 and poly(dA:dT) treatment (Fig. 6D through F), suggesting that IFN-β signaling can be activated by GPX4 activation. In summary, ferroptosis inhibition via GPX4 activation promotes the IRF3 pathway and IFN-β expression, potentially enhancing antiviral immunity.

## DISCUSSION

Ferroptosis is an important defense mechanism of host cells against viral invasion. Various viruses can alter and manipulate ferroptosis during viral infection. In general, most viruses induce ferroptosis to promote replication and facilitate their release from ferroptotic cells, which can lead to host tissue damage and promote pathogen spread. For example, IAV infection has been shown to trigger ferroptosis. Ferroptosis resistance can restrain IAV replication, which significantly impacts host defense against IAV infection (15, 28). Newcastle disease virus increases iron levels and enhances the Fenton reaction by utilizing ferritinophagy to trigger ferroptosis through the p53-SLC7A11-GPX4 pathway, thereby promoting viral replication in tumor cells (32). EBV manipulates ferroptosis during pathogenesis for oncogenic purposes, reducing the sensitivity of nasopharyngeal carcinoma cells to ferroptosis and enhancing their proliferation and chemotherapy resistance (33). Therefore, targeting lipid peroxidation and ferroptosis may represent potential therapeutic strategies for viral infections.

ASFV is a complex enveloped virus with an icosahedral morphology, consisting of four concentric layers and a large double-stranded DNA molecule, ranging in length from approximately 170 to 193 kb between isolates (34). ASFV infection or related proteins can induce oxidative stress (35) and promote ROS accumulation with high levels of 8-oxo-7,8-dihydroguanine and oxoguanine DNA glycosylase 1 (OGG1). Moreover, OGG1 inhibition can activate IFN-β expression to restrict ASFV infection (36), suggesting that inhibiting oxidative damage might be an effective means to control ASFV. The present study demonstrated that ASFV infection reduced cell activity, elevated iron levels, and restricted the keapl1-Nrf2 pathway in PAM cells. Notably, ASFV infection resulted in ROS accumulation and lipid peroxidation, promoting NCOA4-mediated ferritinophagy and ferroptosis. We speculated that the enhancement of ferroptosis in host cells by ASFV might be an important means to evade the host’s innate immunity.

This study aimed to investigate the potential genes of ASFV that participate in the regulation of ferroptosis. MGF505-3R was initially screened for inhibition of GPX4 expression. GPX4 plays a crucial role in maintaining redox homeostasis and functions as a core inhibitor of ferroptosis (37). Subsequently, coimmunoprecipitation experiments were performed to identify the proteins interacting with MGF505-3R in PK-15 cells. Mass spectrometry results and information analysis of GO and KEGG signaling pathway databases showed that MGF505-3R proteins interacted with proteins involved in redox processes, immune system proteins, and catabolic system proteins. Many proteins were involved in GSH metabolism. GARS, a glycyl-tRNA synthetase, is associated with inflammation and cancers (38). Compound heterozygous mutations in GARS cause mitochondrial respiratory chain dysfunction (39). EPRS is a tRNA ligase that is composed of glutamic acid. EPRS inhibition induces the integrated stress response (40, 41). NFS1 is a mitochondrial cysteine desulfurase that restricts ferroptosis in gastric cancer by targeting the STAT3 pathway (42). These proteins are associated with inflammation, stress, and ferroptosis, as well as play a biochemical role in GSH synthesis, and GSH is required for the synthesis of GPX4. To further clarify the role of MGF505-3R in GPX4-mediated ferroptosis, molecular docking, colocalization, and immunoprecipitation were performed to confirm the interaction between MGF505-3R and GPX4. These results further indicated that MGF505-3R transfection facilitated NCOA4-mediated ferritinophagy and downregulated GPX4 expression. These data suggest that MGF505-3R is involved in the regulation of ferroptosis.

Oxidative damage caused by lipid peroxidation is the primary factor triggering ferroptosis. The Keap1-Nrf2 pathway is a classical antioxidant and cytoprotective signaling pathway and is considered a prospective therapeutic target in sickness related to oxidative stress and inflammatory response (17). Nrf2 target molecules, such as FTH1 and GPX4, have been reported to exert antiferroptosis effects. Studies have demonstrated the involvement of Nrf2 in the pathophysiology of viral infections and their associated complications (43). HSV-1 infection enhanced Keap1-mediated ubiquitination and degradation of Nrf2, thereby disturbing cellular redox homeostasis and promoting ferroptosis (22). The H1N1 virus triggered ferroptosis in human nasal epithelial progenitor cells via the Keap1-Nrf2-GCLC pathway, resulting in nasal mucosal epithelial inflammation (23). Tripartite motif-containing protein 21, an E3 ubiquitin ligase, facilitated oxidative stress and ferroptosis by regulating the SQSTM1-Keap1-Nrf2 pathway after highly pathogenic avian influenza virus infection (44). The present study demonstrated that MGF505-3R transfection significantly triggered ROS accumulation and lipid peroxidation, decreased GSH and MMP levels, and restricted the Keap1-Nrf2 pathway. These data suggest that MGF505-3R inhibits the Keap1-Nrf2 axis to suppress antioxidant pathways, thereby promoting oxidative damage and ferroptosis.

Whether ferroptosis caused by MGF505-3R inhibits GPX4 and impedes IFN-β remains to be further clarified. Activation of the cGAS-STING pathway contributes to IFN-β production. This pathway has emerged as a key mediator of the response to infection, stress, tissue damage, and inflammation (45). cGAS senses pathogens or host-derived DNAs and catalyzes the production of cGAMP (46, 47). cGAMP activates STING and further stimulates type I IFN by promoting TBK1, thus facilitating nuclear translocation of the transcription factor IRF3, consequently triggering an antiviral response (46, 48). As a DNA virus, ASFV restricts the production of type I interferon mediated by the cGAS-STING signaling pathway, thereby evading the host’s innate immune response (49, 50). Our results demonstrated that GPX4 activation contributed to the TBK1-IRF3 pathway. These effects were reversed by MGF505-3R treatment. Importantly, GPX4 activation facilitated the expression of IFN-β and ISGs, demonstrating that ferroptosis resistance was conducive to the TBK1-IRF3-mediated IFN-β pathway and promoted antiviral activity.

### Conclusion

**Fig 7:**
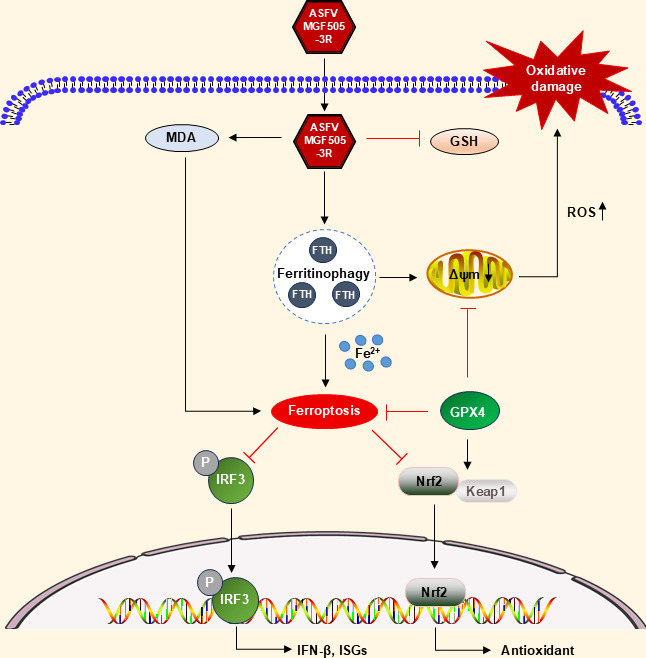
A working model of MGF505-3R promoting ferroptosis to restrict type I IFN production. ASFV infection and MGF505-3R transfection facilitate iron and ROS accumulation, resulting in NCOA4-mediated ferritinophagy and ferroptosis, while the GPX4 and Keap1-Nrf2 pathway is restricted. GPX4 activation promotes the TBK1-IRF3-IFN-β pathway and exerts antivirus capacity.

In summary, our results revealed that ASFV infection and MGF505-3R transfection induced the accumulation of iron and ROS, resulting in NCOA4-mediated ferritinophagy and ferroptosis, as well as restricting GPX4 expression and the Keap1-Nrf2 pathway. GPX4 activation promoted TBK1-IRF3, induced the IFN-β pathway, and exerted antiviral activity (Fig. 7). These findings indicate that ASFV facilitates ferroptosis, providing a proof-of-principle that may be applicable to oxidative damage and lipid peroxidation manipulation-based therapy for ASFV infection. Given the GPX4 downregulation in ASFV infection, GPX4 activation and ferroptosis resistance highlight its potential as a therapeutic target for viral infection.

## Data Availability

The mass spectrometry proteomics data have been deposited to the ProteomeXchange Consortium via the iProX partner repository (51, 52) with the data set identifier PXD064545. Inquiries regarding data sharing can be requested from the corresponding author.

## SUPPLEMENTAL MATERIAL

Supplemental material spectrum.03423-24-s0001.docx Fig. S1 and Table S1.

ASM does not own the copyrights to Supplemental Material that may be linked to, or accessed through, an article. The authors have granted ASM a non-exclusive, world-wide license to publish the Supplemental Material files. Please contact the corresponding author directly for reuse.
