# Supplementary material for: African swine fever virus MGF505-3R facilitates ferroptosis to restrict TBK1-IRF3 pathway
Source: Microbiol Spectr. 2025 Jun 23;13(8):e03423-24. doi: 10.1128/spectrum.03423-24 (PMC12323604; doi:10.1128/spectrum.03423-24)
Supplement: Supplemental material — Fig. S1 and Table S1. [file spectrum.03423-24-s0001.docx]

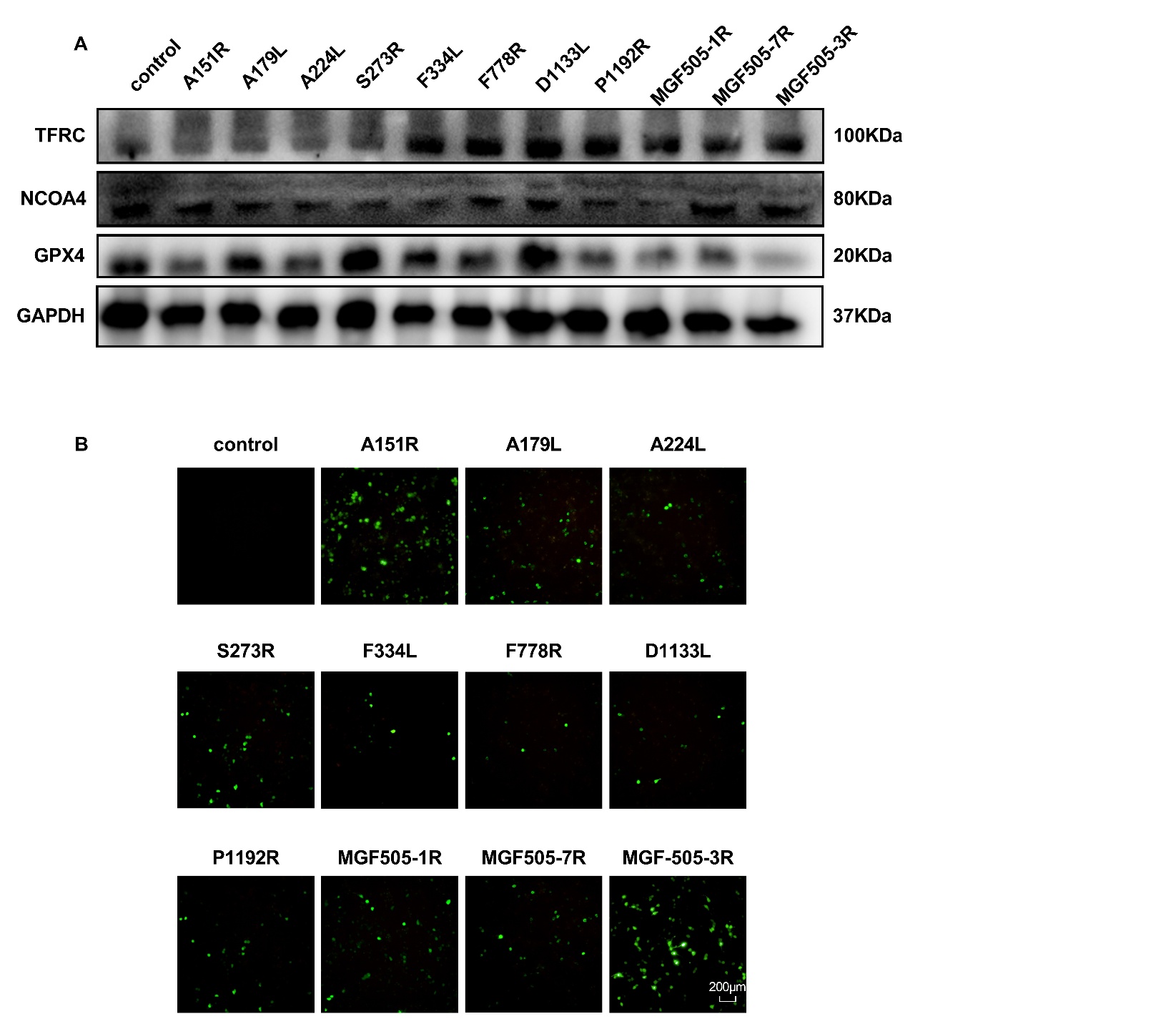


Supplemental Figure S1 (A) Western blot analysis GPX4 expression levels in host cells by transfection with different plasmids. (B) Fluorescence microscope observed the plasmid transfection efficiency.

**Supplemental Table1 Identification of interacting proteins**

| **Protein** | **Description** | **Protein ID** |
| --- | --- | --- |
| LGALS1 | Galectin-1 | NP_001001867.1 |
| ALB | Serum albumin | NP_001005208.1 |
| ANXA2 | Annexin A2 | NP_001005726.1 |
| HSPB1 | Heat shock protein beta-1 | NP_001007519.1 |
| AHCY | Adenosyl-homocysteinase | NP_001011727.1 |
| LOC595122 | Histone H1/5 | NP_001026962.1 |
| RPSA | 40S ribosomal protein SA | NP_001032223.1 |
| TPI1 | Triosephosphate isomerase (TIM) | NP_001032228.1 |
| ATP5O | F-type H+-transporting ATPase subunit O | NP_001038071.1 |
| NME2 | Nucleoside-diphosphate kinase | NP_001038075.1 |
| EEF1A1 | Elongation factor 1-alpha | NP_001090887.1 |
| RPS26 | Small subunit ribosomal protein S26e | NP_001090950.1 |
| KRT2 | Type II keratin, basic | NP_001092053.1 |
| EIF4A2 | Translation initiation factor 4A | NP_001093665.1 |
| RPS20 | Small subunit ribosomal protein S20e | NP_001123426.1 |
| NFS1 | Cysteine desulfurase | NP_001129982.1 |
| RPS16 | Small subunit ribosomal protein S16e | NP_001131092.1 |
| PSMA5 | 20S proteasome subunit alpha 5 | NP_001138373.1 |
| FSCN1 | Fascin 1/2 | NP_001139772.1 |
| CALR | Calreticulin | NP_001167604.1 |
| ATP5A1 | F-type H+-transporting ATPase subunit alpha | NP_001172071.1 |
| RPS4X | Small subunit ribosomal protein S4e | NP_001191212.1 |
| ANXA8 | Annexin A8 | NP_001230528.1 |
| HSPA8 | Heat shock 70kDa protein 1/2/6/8 | NP_001230836.1 |
| MDH2 | Malate dehydrogenase | NP_001231082.1 |
| PFN1 | Profilin | NP_001231345.1 |
| RPS13 | Small subunit ribosomal protein S13e | NP_001231758.1 |
| RAB11B | Ras-related protein Rab-11B | NP_001231804.1 |
| HSPD1 | 60 kDa heat shock protein, mitochondrial | NP_001241645.1 |
| PDIA4 | Protein disulfide-isomerase A4 | NP_001254763.1 |
| RPL29 | Large subunit ribosomal protein L29e | NP_999115.1 |
| RPL35 | Large subunit ribosomal protein L35e | NP_999491.1 |
| RPS15 | Small subunit ribosomal protein S15e | NP_999499.1 |
| YWHAZ | 14-3-3 protein beta/theta/zeta | XP_001927263.3 |
| TUBB4B | Tubulin beta | XP_003122400.2 |
| ACTB | Actin beta/gamma 1 | XP_003124328.1 |
| LOC100525692 | Large subunit ribosomal protein L7Ae | XP_003125024.2 |
| CCT4 | T-complex protein 1 subunit delta | XP_003125148.1 |
| RPS27A | Small subunit ribosomal protein S27Ae | XP_003125184.3 |
| ANXA5 | Annexin A5 | XP_003129266.2 |
| RPS25 | Small subunit ribosomal protein S25e | XP_003129968.1 |
| KRT10 | Type I keratin, acidic | XP_003131508.1 |
| KPNB1 | Importin subunit beta-1 | XP_003131576.3 |
| LOC100516295 | Histone H1/5 | XP_003132451.1 |
| SEPT7 | Septin-7 isoform X1 | XP_003134838.1 |
| GARS | Glycyl-tRNA synthetase | XP_003134854.3 |
| EEF2 | Elongation factor 2 | XP_003354050.1 |
| CCT8 | T-complex protein 1 subunit theta | XP_003358947.2 |
| RPL8 | Large subunit ribosomal protein L8e | XP_005655354.1 |
| MYL6 | Myosin light chain 6 | XP_005655644.1 |
| RPL4 | Large subunit ribosomal protein L4e | XP_005659919.2 |
| LGALS3 | Galectin-3 | XP_005660031.1 |
| EEF1G | Elongation factor 1-gamma | XP_005660855.3 |
| HSPA9 | Stress-70 protein, mitochondrial | XP_005661752.1 |
| RPL31 | 60S ribosomal protein L31 | XP_005662419.1 |
| HSP90AB1 | Heat shock protein HSP 90-beta isoform | XP_005666120.1 |
| VIM | Vimentin | XP_005668163.1 |
| YWHAB | 14-3-3 protein beta/theta/zeta | XP_005673018.1 |
| RPL9 | Large subunit ribosomal protein L9e | XP_013834051.1 |
| LOC100127131 | Tubulin alpha | XP_013843702.1 |
| EZR | Radixin | XP_013847913.2 |
| LDHA | L-lactate dehydrogenase | XP_013849955.2 |
| HYOU1 | Hypoxia up-regulated 1 | XP_020918615.1 |
| EPRS | bifunctional glutamate/proline--tRNA ligase isoform | XP_020919973.1 |
| ALDH1A1 | Retinal dehydrogenase | XP_020920570.1 |
| P4HB | Protein disulfide isomerase family A, member 2 | XP_020922086.1 |
| LOC110255312 | Type I keratin, acidic | XP_020922659.1 |
| HSPA5 | Heat shock 70kDa protein 5 | XP_020924489.1 |
| NCL | Nucleolin isoform | XP_020930618.1 |
| RPL37A | Large subunit ribosomal protein L37Ae | XP_020931904.1 |
| SDHA | Succinate dehydrogenase flavoprotein subunit | XP_020932589.1 |
| NNT | NAD(P) transhydrogenase | XP_020932660.1 |
| LOC102162486 | RNA-binding protein Luc7-like 2 | XP_020934505.1 |
| UBA1 | Ubiquitin-activating enzyme E1 | XP_020936014.1 |
| RPS2 | Small subunit ribosomal protein S2e | XP_020942455.1 |
| ALDOA | Fructose-bisphosphate aldolase, class I | XP_020943654.1 |
| RPL30 | Large subunit ribosomal protein L30e | XP_020944244.1 |
| GAPDH | Glyceraldehyde 3-phosphate dehydrogenase | XP_020946773.1 |
| NACA | nascent polypeptide-associated complex subunit alpha | XP_020947462.1 |
| ENO1 | Enolase | XP_020950938.1 |
| SRSF3 | Splicing factor, arginine/serine-rich 3 | XP_020954304.1 |
| PKM | Pyruvate kinase | XP_020954765.1 |
| RAB15 | Ras-related protein Rab-15 | XP_020955042.1 |
| LOC100525821 | Histone H3 | XP_020955511.1 |
| LOC100624785 | Tubulin beta | XP_020955779.1 |
| PDLIM5 | LIM domain-binding protein 3 | XP_020957405.1 |
